# Crystal structure and function of Rbj: A constitutively GTP-bound small G protein with an extra DnaJ domain

**DOI:** 10.1007/s13238-019-0622-3

**Published:** 2019-04-03

**Authors:** Zhengrong Gao, Keke Xing, Chang Zhang, Jianxun Qi, Liang Wang, Shan Gao, Ren Lai

**Affiliations:** 1 Key Laboratory of Bioactive Peptides of Yunnan Province/Key Laboratory of Animal Models and Human Disease Mechanisms of Chinese Academy of Sciences, Kunming Institute of Zoology, Kunming, 650223 China; 2 CAS Key Laboratory of Bio-medical Diagnostics, Suzhou Institute of Biomedical Engineering and Technology, Chinese Academy of Sciences, Suzhou, 215163 China; 3 University of Chinese Academy of Sciences, Beijing, 100049 China; 4 CAS Key Laboratory of Pathogenic Microbiology and Immunology, Institute of Microbiology, Chinese Academy of Sciences, Beijing, 100101 China

**Dear Editor,**

The Ras superfamily of GTPases are conserved in eukaryotes from yeast to humans, and play essential roles in the regulation of a variety of key cellular processes, including cell differentiation and proliferation, membrane trafficking, nuclear import and export, cytoskeletal remodeling and mitogenic signaling (Karnoub and Weinberg, 2008). Notably, numerous studies have shown that many members of the Ras-related small GTPases are involved in diverse aspects of tumorigenesis and tumor progression (Chen et al., 2014; Liu et al., 2017). These functions are a consequence of the GTP hydrolysis reaction catalyzed by a GTPase-domain (G-domain), which can bind to and hydrolyze guanosine triphosphate (GTP) (“on” state) to guanosine diphosphate (GDP) (“off” state) . However, a small number of proteins naturally do not have the ability to hydrolyze GTP, yet play an important role in some diseases with unknown mechanisms (Chardin, 1999; Fiegen et al., 2002). To date, the Ras superfamily contains over 150 members, which can be divided into six subfamilies: Ras, Rho, Rab, Arf, Ran and RJL, based on sequence homology, structure and function (Goitre et al., 2014). The RJL family is an independent lineage of the Ras superfamily of GTPases, which can be classified into two distinct subfamilies: Rjl and Rbj (Nepomuceno-Silva et al., 2004). Besides the G-domain, members in this family contain an extra DnaJ domain. However, the function-structure relationship of these two domains and the function of the DnaJ domain are yet unclear.

To understand the molecular basis behind their interactions of the latest identified small GTPase family members, the *Xenopus laevis* Rbj (xRbj) protein was expressed in *Escherichia coli* and its crystal structure has been determined to a resolution of 2.7 Å (Table S1). Two molecules were observed in one crystallographic asymmetric unit. While the 13 residues at the N-terminus are flexible, so each molecule contains 260 residues, spanning A14 to K273. The structure reveals that the G-domain and DnaJ domain are two independent domains, connected by a flexible hinge region (Fig. 1), thereby providing enough flexibility for binding effector proteins independently. Superimposing the G-domain of the two molecules in the asymmetric unit indicates that the two DnaJ domains are pointing to different directions, branching via the hinge region (Fig. S1), confirming the flexibility of the hinge region. The xRbj displays a typical GTPase fold consisting of a six-stranded β sheet (five parallel and one antiparallel) surrounded by five α helices, a typical Ras structure (Kityk et al., 2018) (Fig. S2A). One additional small helix is present between the α4 helix and β5 strand (Fig. 1B and 1C). The DnaJ domain consists of four α helices (Fig. 1B and 1C). In the GTPase active site, the Mg^2+^ ion is tightly coordinated by the conserved residues S30 in P-loop, T48 in switch I, the γ-phosphate and β-phosphate atoms of the nucleotide and two well-ordered water molecules (Scheidig et al., 1999) (Fig. S2B). Unlike the strong GTPase activity of the Ras protein, xRbj remains in an “on” state, which has little, if any, GTPase activity (Fig. 2D). Previous studies indicated that Q61 in the switch II loop of Ras plays a key role in the intrinsic GTP hydrolysis reaction (Novelli et al., 2018) (Fig. 2A). Mutations of Ras at Q61 are known to slow the rate of GTP hydrolysis and transform healthy cells into malignant cells (Bos, 1989). The following residue E63 further stabilizes the position and orientation of Q61 by a hydrogen bond between the side chains (Fig. 2B). In contrast, while the counterpart residue H75 in xRbj possesses similar side chain length as Q61, the H75 side chain is oriented in the opposite direction of GTP, forming a hydrogen bond with the main chain of F77 (Fig. 2B). P76 is also more rigid than E62 in Ras, which further stabilizes the switch II loop. Consequently, H75 orients away from the γ-phosphate, which results in abrogation of GTPase activity (Fig. 2B). Therefore, we have defined xRbj G-domain as a GTP-bound protein.

**Figure 1:**
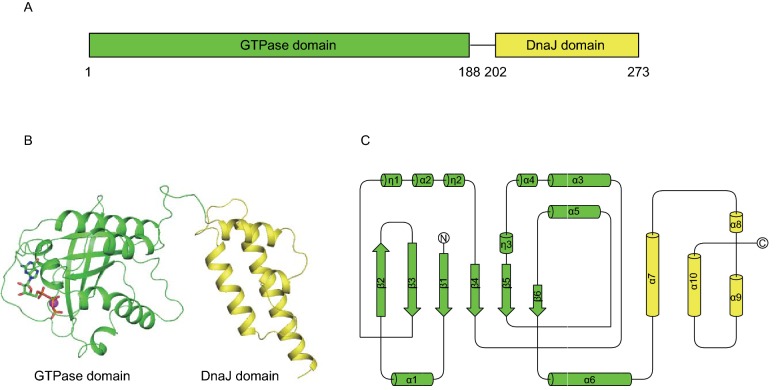
**Crystal structure of xRbj**. (A) Schematic picture of xRbj. The GTPase domain and DnaJ domain are shown in green and yellow, respectively. (B) Cartoon representation of the crystal structure. The GTPase domain and DnaJ domain are colored the same as Fig. 1A. GTP is displayed in stick format and the Mg^2+^ cation is represented as a magenta sphere. (C) Topology diagram of the GTPase domain and DnaJ domains

**Figure 2:**
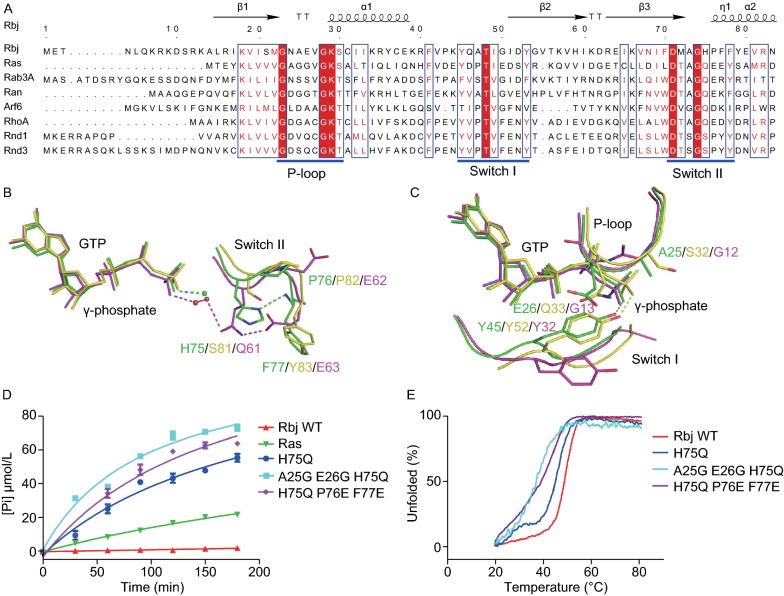
**The structural and functional analysis of GTP binding sites**. (A) Sequence alignment of the GTP binding sites among representative members of six small GTPase families, with hydrolysis activity (Ras, Rab3A, Ran, Arf6, RhoA) and without activity (Rnd1, Rnd3). The highly conserved amino acids are marked in red. Three GTP binding motifs (P-loop, switch I loop and switch II loop) are highlighted in the blue bar below the sequence. Database sequence accession numbers: Rbj (Q72YF1), Ras (P01112), Rab3A (P20336), Ran (P62826), Arf6 (P62330), RhoA (P61586), Rnd1 (Q92730), Rnd3 (P61587). (B) The structural alignment of key residues on the switch II loop among xRbj (green), Ras (magenta) and Rnd3 (yellow). The amino acids and GTP are displayed in stick format, while the switch loop is displayed in cartoon format. (C) Structural alignment of key residues on the P-loop and switch I loop among xRbj (green), Ras (magenta) and Rnd3 (yellow). The amino acids and GTP are displayed in sticks format, while the loops are shown in cartoon format. The PBD for xRbj, Ras and Rnd3 are 6JMG, 1QRA and 1M7B, respectively. (D) Enzyme activities of xRbj wildtype (red), Ras (green), xRbj-H75Q (blue), xRbj-A25GE26GH75Q (cyan) and xRbj-H75QP76EF77E (purple). (E) Thermostability analysis of the xRbj wildtype and three xRbj mutant proteins. The colors are consistent with Fig. 2D

Site-directed mutagenesis experiments were performed to examine in more detail the contributions of the different residues to GTPase activity. H75Q (xRbj-H75Q) increased the GTPase activity of xRbj (Fig. 2D), which is consistent with previous reports (Chen et al., 2014). Triple mutations (xRbj-H75Q-P76E-F77E) on the switch II loop designed according to the structural analysis increased the hydrolysis activities to a greater extent, suggesting that these three residues are important for the lack of GTPase activity in xRbj. Notably, the serine in two small GTPases with defective GTPase activities (S71 in Rnd1 and S81 in Rnd3) (Chardin, 1999; Fiegen et al., 2002), which corresponds to H75 in xRbj on the switch II loop, also deviates from the γ-phosphate of GTP. Moreover, the serine (S81 in Rnd3) side chain is shorter than the glutamine (Q61 in Ras), and thus is unable to sufficiently stabilize the transient hydronium ion (Fiegen et al., 2002). Consistent with P76 in xRbj, the corresponding residues are both prolines (P72 in Rnd1 and P82 in Rnd3), suggesting similar reasons for lack of GTPase activity (Fig. 2B). Aside from Q61, residues G12 and G13 in Ras are also important to GTPase activity. Mutations of these two residues impair GTP hydrolysis and result in tumorigenesis (Ostrem et al., 2013). G12 and G13 are located on the P-loop of Ras (Fig. 2A). The short nature of the glycine side chains enables an “open” conformational state in Ras. In xRbj the corresponding site contains A25 and E26, which displays a “closed” GTP binding site due to the bulkier nature of the side chains, and the E26 side chain sterically limits the space in the binding site (Fig. 2C). Additionally, a tyrosine residue on the switch I loop also contributes to the GTP binding site. Specifically, Y45 in the P-loop of xRbj holds the oxygen of the GTP γ-phosphate, while Y32 in the corresponding position of Ras points outside of the pocket. Interestingly, both Rnd1 and Rnd3 contain residues with similar length in this region, and the side chain of tyrosine on the switch I loop display parallel orientation to the Y45 in xRbj (Fig. 2C). To test whether these residues would contribute to GTPase activity in xRbj, a GTPase enzyme activity assay was performed which demonstrates that deletion of the side chains of A25 and E26 combined with H75Q (xRbj-A25G-E26G-H75Q) substantially increases the GTPase activity compared to H75Q alone (Fig. 2D). A thermostability assay was subsequently performed which indicates that wildtype xRbj is more stable than mutant xRbjs (Fig. 2E), suggesting that xRbj prefers to bind GTP and that this may be important for its stability *in vivo*, thereby xRbj might only be a GTP-bound G-protein with undefined function, which needs further studies.

The DnaJ domain-containing protein chaperone, also known as Hsp40, is expressed in a wide variety of organisms from bacteria to humans (Kityk et al., 2018). To date, RJL family members are the only Ras superfamily members containing an extra DnaJ domain (Elias and Archibald, 2009). However, the structure and function of the DnaJ domain in the Ras superfamily remains unknown. Our xRbj structure displays the DnaJ molecular arrangement for the first time. Sequence alignment analysis shows that the identity of Rbj-DnaJ domain, spanning 203 to 273, in different species is 67.7% (Fig. S3). All of them contain a highly conserved HPD loop, which is the feature of the “J” domain-containing proteins (Elias and Archibald, 2009) (Fig. S3). The sequence identity of the Rbj-DnaJ domain and DnaJ proteins with known structures in other species is 48.3%. Structural comparison of the xRbj-DnaJ domain with the orthologues in other species, shows that human Rbj (residues 1–89, corresponding residues 203–273 in xRbj) displays the most similarity with RMSD of 0.75 Å, whereas DnaJ in *E*. *coli* (residues 2–76, corresponding residues 203–273 in xRbj) shares the lowest similarity with RMSD of 1.41 Å. Previous reports suggest that DnaJ proteins interact with another important chaperone, the DnaK proteins (also called Hsp70). Further structural studies demonstrated that DnaJ binds to the substrate polypeptide-binding domain (SBD) and nucleotide-binding domain (NBD) of Hsp70, which is a prerequisite for the activation of Hsp70 (Karzai and McMacken, 1996) (Fig. S4A). Interestingly, the superimposition of the two structures indicates that the GTPase domain exerts no steric hindrance on the association with Hsp70 (Kityk et al., 2018) (Fig. S4B). Structural analysis shows that twelve key residues are responsible for binding to Hsp70. Ten of them exert similar properties, and six of these are conserved in DnaJ domains and Rbjs in different species. Previous studies suggested that human Rbj is an oncogenic small GTPase required for gastrointestinal cancer tumorigenesis and progression by mediating nuclear accumulation of active mitogen-activated protein kinase kinase 1/2 (MEK1/2) and sustained activation of extracellular signal-regulated kinase 1/2 (ERK1/2) (Chen et al., 2014). The C-terminal DnaJ domain of Rbj could potentially directly interact with both MEK1/MEK2 and ERK1/ERK2 (Chen et al., 2014). Previous studies show that DnaJ is a molecular chaperone and belongs to the J-protein family which can regulate the activity of 70-kDa heat-shock proteins (Walsh et al., 2004). The DnaJ domain of Rbj also belongs to the J-protein family, therefore, Hsp70 may also be one of the effector proteins of Rbj.

In summary, we determined the first structure of small GTPase xRbj containing a typical GTP-binding domain and an extra DnaJ domain. Both domains are folded independently and linked by a highly-flexible hinge region. The structure suggests that xRbj lacks intrinsic GTPase activity due to the features of the three nucleotide-binding regions of the G-domain, which was subsequently confirmed by mutagenesis studies. The mode of interaction between xRbj and Hsp70 provides a possible binding mechanism between DnaJ domain and effector protein.

## Electronic supplementary material

Below is the link to the electronic supplementary material.

Supplementary material 1 (PDF 771 kb)
