# Supplementary material for: Crystal structure and function of Rbj: A constitutively GTP-bound small G protein with an extra DnaJ domain
Source: Protein Cell. 2019 Apr 3;10(10):760–3. doi: 10.1007/s13238-019-0622-3 (PMC6776491; doi:10.1007/s13238-019-0622-3)
Supplement: Supplementary file 1 — Supplementary material 1 (PDF 771 kb) [file 13238_2019_622_MOESM1_ESM.pdf]

Supplemental Files

Crystal structure and function of Rbj: A constitutively GTP-bound small G protein with an extra DnaJ domain

Zhengrong Gao[1,3], Keke Xing[2], Chang Zhang[2], Jianxun Qi[4], Liang Wang[4], Shan Gao[2,*], Ren Lai[1,*]

[1] Key Laboratory of Bioactive Peptides of Yunnan Province/Key Laboratory of Animal Models and Human Disease Mechanisms of Chinese Academy of Sciences, Kunming Institute of Zoology, Kunming 650223, Yunnan Province, China.

[2] CAS Key Laboratory of Bio-medical Diagnostics, Suzhou Institute of Biomedical Engineering and Technology, Chinese Academy of Sciences, Suzhou 215163, Jiangsu Province, China.

[3] University of Chinese Academy of Sciences, Beijing 100049, China.

[4] CAS Key Laboratory of Pathogenic Microbiology and Immunology, Institute of Microbiology, Chinese Academy of Sciences, Beijing 100101, China.

*Corresponding authors:

Prof. Ren Lai, No. 32, Jiaochang East Road, Wuhua District, Kunming 650223, Yunnan Province, China, 86-871-65197578 (Tel.), 86-871-65197578 (Fax), E-mail: rlai@mail.kiz.ac.cn

Prof. Shan Gao, No. 88, Keling Road, Science and Technology City, High-tech Zone,

Suzhou 215163, China, 86-0512-69588000 (Tel.), 86-0512-69588000 (Fax), E-mail:

gaos@sibet.ac.cn

Materials and Methods

Construction of Rbj expression vectors.

The full-length *X. laevis Rbj* gene (*xRbj*, residues 1-273) was synthesized based on *GenBank* entry NC_Q7ZYF1 and codon optimized for expression in *E.coli*. The gene was cloned into the expression vector pET21a to form *Rbj*-pET21a, with a 6xHis tag at the C-terminus. For protein expression, the *Rbj*-pET21a plasmid was transformed into *E. coli* BL21 (DE3) competent cells. H75Q, A25G-E26G-H75Q, H75Q-P76E-F77E mutants were constructed based on *Rbj*-pET21a and expressed in the same way.

Expression and purification of Rbj proteins.

E. coli BL21 (DE3) cells carrying the *Rbj*-pET21a were grown in LB medium at 37℃ containing 50 mg/L ampicillin sodium. When the cell culture reached an optical density of A_{600}=0.8, 0.3 mM isopropyl-β-D-thiogalactoside (IPTG) was added to induce protein expression. The cells were harvested 16 h post induction. The bacterial pellet was re-suspended by adding lysis buffer A (20 mM Tris pH 8.0, 500 mM NaCl, 5% glycerol, 1 mM $MgCl_2$) and then lysed by passing through a microfluidizer twice. The supernatant containing the soluble Rbj protein was passed through a 5 mL Ni-NTA column (GE Healthcare) pre-equilibrated with lysis buffer A for 1 h at 4℃. The column was subsequently washed with buffer B (20 mM Tris pH 8.0, 500 mM NaCl, 5% glycerol, 20 mM imidazole, 1 mM $MgCl_2$) for 20 column volumes. The Rbj protein was eluted with a solution of 300 mM imidazole, 20 mM Tris pH 8.0, 500 mM

NaCl, 5% glycerol, 1 mM $MgCl_2$. The eluted Rbj protein was pooled and loaded onto a Superdex® 75 16/60 column (GE Healthcare), which was equilibrated with buffer C (20 mM Tris pH 8.0, 150 mM NaCl, 1 mM $MgCl_2$, 1 mM DTT). The resulting protein peak was concentrated to 15 mg/ml using a 30 kDa MWCO Amicon® Ultra centrifugal concentrator. Aliquots were snap-frozen in liquid nitrogen and stored at -80℃ until they were used for crystallization. All the Rbj mutant proteins were prepared in the same way as the wildtype Rbj.

Crystallization and structural determination of Rbj.

For initial trial, Rbj was crystallized at 16℃ with vapor diffusion method in sitting drops. A drop of 1.3 μL of protein at a concentration of 15 mg/ml was mixed with 1.3 μL of precipitation solution consisting of 0.1 M Bis-Tris pH 6.5, 28% (w/v) Polyethylene glycol monomethyl ether 2000. Initial crystals formed within 5 days. After optimization, the most optimal crystal formed under 0.1 M Bis-Tris pH 6.0/6.2, 26% (w/v) Polyethylene glycol monomethyl ether 2000. Flash freezing in liquid nitrogen was performed in the same solution with 20% glycerol as cryoprotectant. Data were collected at the Shanghai Synchrotron Radiation Facility (SSRF) BL19U. Data were processed with HKL2000 (Otwinowski and Minor, 1997). The structure of Rbj was determined by molecular replacement with homologous structures (PDB 2A5J and 2YS8) as search models using the program Phaser (Read, 2001). The atomic model was rebuilt and completed with Coot (Emsley and Cowtan, 2004) and refined with phenix.refine (Adams et al., 2010). The stereochemical quality of the

final model was assessed with PROCHECK (Laskowski R, 1993). Data collection and refinement statistics are summarized in Table S1. All structural figures were generated with PyMol (http://pymol.org). The atomic coordinate has been deposited in the Protein Data Bank. The PDB ID code is 6JMG.

Intrinsic GTPase activity essay

The intrinsic GTPase activity was monitored under the single-turnover condition adapting a strategy similar to Chandra *et al* (Chandra et al., 2014). The activity of both mutant and wildtype Rbj was evaluated under the concentration of 400 μM, with addition of 95 mM GTP in a buffer of 20 mM Tris pH 8.0, 150 mM NaCl, 10 mM EDTA, 2 mM DTT. The reaction was incubated for 90 minutes at 25 ℃. The excess unbound nucleotides were subsequently removed via desalting chromatography (NAP-5, GE Healthcare Life Sciences). The samples were eluted with 200 μL buffer.

An ATPase/GTPase activity assay kit (Sigma, Cat No. MAK113) was used to determine the enzyme activity. The 68 μM nucleic acid-bound protein was incubated with 20 mM $MgCl_2$ at 37℃. For 0 min, 30 min, 60 min, 90 min, 120 min, 150 min and 180 min according to the experimental requirements. The reaction was stopped by addition of 200 μL stop solution and incubated at room temperature for another 30 min. The output values were recorded using a microplate reader and used to calculate GTPase activity.

Circular dichroism (CD) spectroscopy.

The thermostability of wild type and mutant Rbj was tested by CD spectroscopy. The protein concentration was 0.2 mg/ml in 20 mM Tris (pH 8.0), 150 mM NaCl, 1mM $MgCl_2$ and 1mM DTT. Temperature-dependent CD experiments were analyzed at 205-260 nm from 20-80℃ with intervals of 1℃ to determine melting temperature (*Tm*). The data was analyzed using Prism 5 software.

Supplementary Table 1. Statistics for crystallographic data collection and structure refinement.

**Data collection statistics**	
Wavelength (Å)	0.97910
Resolution range (Å)	50.0-2.70 (2.80-2.70) *
Space group	P $6_1$22
Unit cell dimensions	
a, b, c (Å)	107.63, 107.63, 320.25
α, β, γ (°)	90.00, 90.00, 120.00
Unique reflections	31168 (3009) *
Completeness (%)	99.9 (100)
Mean I/sigma(I)	30.1 (2.5)
Redundancy	19.6 (20.7)
Rmerge (%)	10.8 (146.5)
Rpim (%)	2.5 (32.8)
CC1/2	0.999 (0.808）
Wilson B-factor	72.4
**Structure refinement**	
Resolution (Å)	50.00–2.70
R_{work} / R_{free} (%)	20.83/25.45
No. atoms	
Protein	4206
Ligands	66
Water	14
RMSD	
Bond lengths (Å)	0.009
Bond angles (°)	1.19
Average B-factors	79.5
Ramachandran plot	
Favored (%)	96.20
Allowed (%)	3.80
Outliers (%)	0

*Values in parentheses are given for the highest resolution shell.

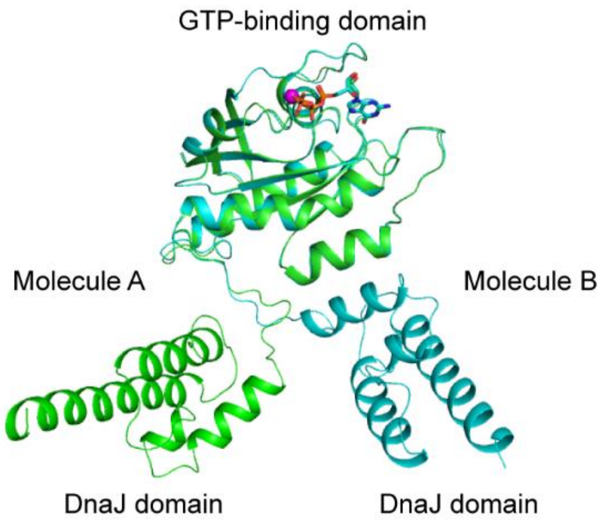

Supplemental Figure 1. Superimposition of the G-domain of two xRbj molecules in the asymmetric unit. Molecule A and B are indicated in green and cyan cartoon representation, respectively. GTP molecules are shown in sticks and Mg^{2+} cations are displayed in spheres. Clearly, DnaJ domain displays two different positions.

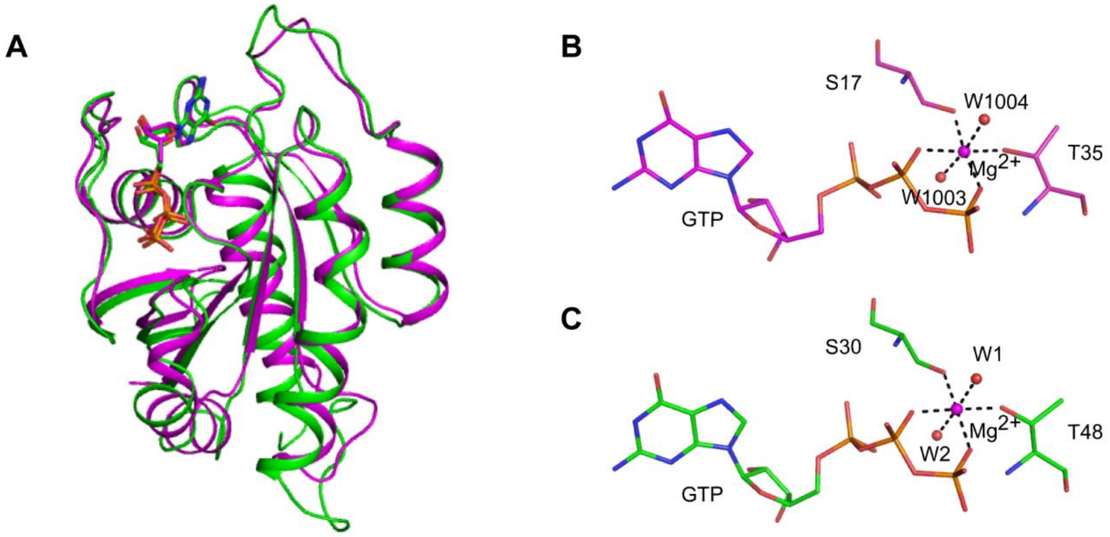

Supplemental Figure 2. The overall structures of G-domain between xRbj and Ras. A. Superimposition of G-domain between xRbj (green) and Ras (magenta) in cartoon mode. B. The coordination of magnesium ions in xRbj and Ras is similar. The GTP and residues are displayed in sticks. The water molecules and the Mg^{2+} are indicated in red sphere and magenta sphere, respectively.

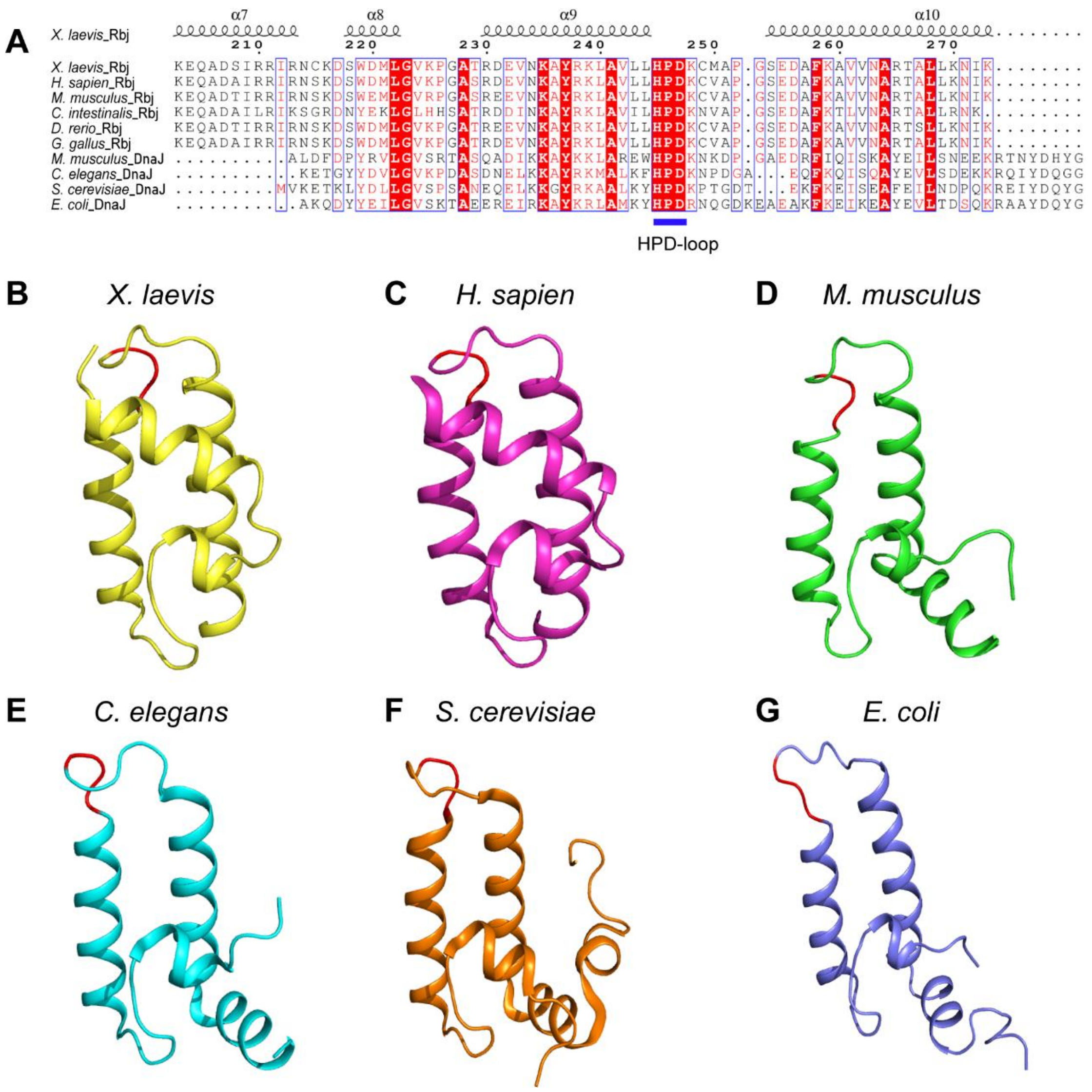

Supplemental Figure 3. Sequences alignment and structural comparison for DnaJ domain. (A) Sequences alignment of the DnaJ domain of Rbj proteins, as well as DnaJ proteins in different species. Sequences used in the alignment are the followings: *X. laevis*_Rbj, Q7ZYF1; *H. sapien*_Rbj, Q9NZQ0; *M. musculus*_Rbj, Q8CFP6; *D. rerio*_Rbj, Q6IMK3; *C. intestinalis*_Rbj, Q6IMM2; *G. gallus*_Rbj, Q6IMM1; *M. musculus*_DnaJ, Q80TN4; *C. elegans*_DnaJ, O45502; *S. cerevisiae*_DnaJ, P25294; *E.coli*, P08622.(B-G) Structural comparisons between the DnaJ domain in xRbj and different species deposited in the PDB database. The accession numbers are the followings: *H. sapien*, 2YS8; *M. musculus*, 2CUG; *C.*

elegans: 2OCH; *S. cerevisiae*, 4RWU; *E. coli*, 1XBL. Each DnaJ domain is displayed in cartoon. Red highlights the conserved HPD loop.

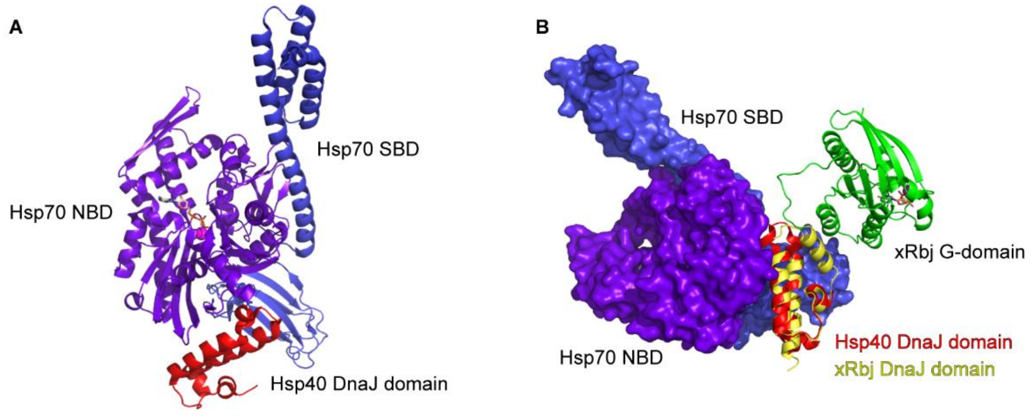

Supplemental Figure 4. The interaction between Hsp70 and DnaJ domain and the prediction of binding mode of Hsp70 to xRbj. (A) Cartoon representation of the complex structure of Hsp70 and DnaJ domain (PDB code: 5NRO). The substrate polypeptide-binding domain (SBD) and nucleotide-binding domain (NBD) in Hsp70 are shown in blue and purple, respectively. The DnaJ domain is exhibited in red. (B) Superimposition of the complex structure of Hsp70-DnaJ domain with xRbj. Hsp70 is shown in surface representation and colored the same as in Supplemental Figure 4A. The DnaJ domain in xRbj is colored in yellow, while the GTPase domain is highlighted in green. The Mg^{2+} and nucleotide moiety are indicated in magenta sphere and pink sticks, respectively.

Adams, P.D., Afonine, P.V., Bunkoczi, G., Chen, V.B., Davis, I.W., Echols, N., Headd, J.J., Hung, L.W., Kapral, G.J., Grosse-Kunstleve, R.W., *et al.* (2010). PHENIX: a comprehensive Python-based system for macromolecular structure solution. Acta Crystallogr D Biol Crystallogr 66, 213-221.
Chandra, M., Mukherjee, M., Srivastava, V.K., Saito-Nakano, Y., Nozaki, T., and Datta, S. (2014). Insights into the GTP/GDP cycle of RabX3, a novel GTPase from Entamoeba histolytica with tandem G-domains. Biochemistry 53, 1191-1205.
Emsley, P., and Cowtan, K. (2004). Coot: model-building tools for molecular graphics. Acta Crystallogr D Biol Crystallogr 60, 2126-2132.
Laskowski R, M.M., Moss D, Thornton J (1993). PROCHECK: A program to check the stereochemical quality of protein structures. J Appl Cryst 26, 283-291.
Otwinowski, Z., and Minor, W. (1997). [20] Processing of X-ray diffraction data collected in oscillation

mode. Methods Enzymol 276, 307-326.
Read, R.J. (2001). Pushing the boundaries of molecular replacement with maximum likelihood. Acta Crystallogr D Biol Crystallogr 57, 1373-1382.
